# TRPC3, but not TRPC1, as a good therapeutic target for standalone or complementary treatment of DMD

**DOI:** 10.1186/s12967-021-03191-9

**Published:** 2021-12-20

**Authors:** Anna Creisméas, Claire Gazaille, Audrey Bourdon, Marc-Antoine Lallemand, Virginie François, Marine Allais, Mireille Ledevin, Thibaut Larcher, Gilles Toumaniantz, Aude Lafoux, Corinne Huchet, Ignacio Anegon, Oumeya Adjali, Caroline Le Guiner, Bodvaël Fraysse

**Affiliations:** 1 Nantes Gene Therapy Laboratory, Université de Nantes, INSERM UMR 1089, IRS 2 Nantes Biotech, CHU de Nantes, 22, Boulevard Bénoni Goullin, 44200 Nantes, France; 2 INRAE, ONIRIS, PAnTher, Nantes, France; 3 L’Institut du Thorax, Université de Nantes, CNRS, INSERM UMR 1087, Nantes, France; 4 Therassay Platform, Capacités, Université de Nantes, Nantes, France; 5 INSERM, UMR 1064-Center for Research in Transplantation and Immunology, ITUN, CHU Nantes, Université de Nantes, Faculté de Médecine, Nantes, France

**Keywords:** DMD, Calcium, TRPC1, TRPC3, *DMD*^*mdx*^ rat, Gene therapy, Skeletal muscle

## Abstract

**Background:**

Duchenne muscular dystrophy (DMD) is an X-linked inherited disease caused by mutations in the gene encoding dystrophin that leads to a severe and ultimately life limiting muscle-wasting condition. Recombinant adeno-associated vector (rAAV)-based gene therapy is promising, but the size of the full-length dystrophin cDNA exceeds the packaging capacity of a rAAV. Alternative or complementary strategies that could treat DMD patients are thus needed. Intracellular calcium overload due to a sarcolemma permeability to calcium (SPCa) increase is an early and critical step of the DMD pathogenesis. We assessed herein whether TRPC1 and TRPC3 calcium channels may be involved in skeletal muscle SPCa alterations and could represent therapeutic targets to treat DMD.

**Methods:**

All experiments were conducted in the *DMD*^*mdx*^ rat, an animal model that closely reproduces the human DMD disease. We measured the cytosolic calcium concentration ([Ca^2+^]_c_) and SPCa in EDL (*Extensor Digitorum Longus*) muscle fibers from age-matched WT and *DMD*^*mdx*^ rats of 1.5 to 7 months old. TRPC1 and TRPC3 expressions were measured in the EDL muscles at both the mRNA and protein levels, by RT-qPCR, western blot and immunocytofluorescence analysis.

**Results:**

As expected from the malignant hyperthermia like episodes observed in several *DMD*^*mdx*^ rats, calcium homeostasis alterations were confirmed by measurements of early increases in [Ca^2+^]_c_ and SPCa in muscle fibers. TRPC3 and TRPC1 protein levels were increased in *DMD*^*mdx*^ rats. This was observed as soon as 1.5 months of age for TRPC3 but only at 7 months of age for TRPC1. A slight but reliable shift of the TRPC3 apparent molecular weight was observed in *DMD*^*mdx*^ rat muscles. Intracellular localization of both channels was not altered. We thus focused our attention on TRPC3. Application of Pyr10, a specific inhibitor of TRPC3, abolished the differences between SPCa values measured in WT and *DMD*^*mdx*^. Finally, we showed that a rAAV-microdystrophin based treatment induced a high microdystrophin expression but only partial prevention of calcium homeostasis alterations, skeletal muscle force and TRPC3 protein increase.

**Conclusions:**

All together our results show that correcting TRPC3 channel expression and/or activity appear to be a promising approach as a single or as a rAAV-based complementary therapy to treat DMD.

## Introduction

Duchenne muscular dystrophy (DMD) is an X-linked inherited disease affecting ~ 1:5000 male births and leading to a severe, highly debilitating and ultimately life limiting muscle-wasting condition [1]. DMD is caused by mutations in the gene encoding dystrophin, a critical protein for the stability and function of skeletal myofibers and cardiomyocytes [2, 3]. Dystrophin establishes a mechanical link between the actin cytoskeleton and the extracellular matrix in muscle fibers through the dystrophin-associated protein complex. DMD-affected boys develop muscle weakness during the first years of life. During teenagehood, they generally become wheelchair-bound and exhibit life-threatening complications caused by respiratory muscle wasting and dilated cardiomyopathy. DMD patients rarely survive into their fourth decade [4]. Gene therapy to restore dystrophin expression is a promising approach for the treatment of DMD. Recombinant adeno-associated virus (rAAV) vectors are particularly efficient in transducing skeletal muscle fibers and cardiomyocytes when packaged with the appropriate capsid [5–7], and allow long-term in vivo transgene expression [8]. However, the full-length dystrophin complementary DNA (cDNA) is 14 kb in length and greatly exceeds the packaging capacity of a single rAAV vector (< 5 kb) [9]. Therefore, shortened transgenes, coding for partially functional microdystrophins (MD) that contain essential domains of the dystrophin protein have been generated. The principle of using MDs as therapeutic transgenes arose from the concept that Becker Muscular Dystrophy (BMD) patients with natural in-frame deletions/mutations in their *DMD* gene exhibit a milder dystrophinopathy [10]. Our group participated in the first study describing long-term functional rescue after a gene therapy treatment based on rAAV-MD systemic delivery in the Golden Retriever Muscular Dystrophy (GRMD) dog, a large animal model of DMD [11]. Three clinical trials using this strategy have been launched in 2018, and the first reported results are very promising [12]. In BMD patients, the disease is milder and more heterogeneous compared to DMD patients. Nevertheless, muscle weakness is often noticed in adolescence or young adulthood [13]. Additionally, current rAAV-MD trials are based on MDs that are ~ 40% smaller than the smallest naturally truncated dystrophin reported in a patient with BMD [14]. It is thus urgent to find alternative or complementary therapeutic strategies for MD-based gene therapy that could treat both DMD and BMD patients. Such strategies have to target significant and primordial events of the DMD pathogenesis.

Calcium plays a critical role in the pathogenesis of DMD as skeletal muscle necrosis is mainly caused by intracellular Ca^2+^ overload [15]. Calcium alterations are very early events: they have been detected in muscle fibers of DMD boy fetuses and measured in not fully differentiated human DMD myotubes [16, 17]. Intracellular Ca^2+^ overload in DMD is mainly related to an increase of the sarcolemma permeability to Ca^2+^ (SPCa) through the accumulation of Ca^2+^ permeable ion channels [18]. The identity of the channels involved in the SPCa increase is still unclear but members of the Transient Receptor Potential (TRP) family have been proposed as possible candidates [18]. The mammalian TRP channel superfamily encompasses 28 members that are subdivided into 6 subfamilies according to their sequence homology [19]. Two TRP channels caught our attention in the context of the DMD: TRPC1 and TRPC3. The expression of these channels is increased in skeletal, cardiac and smooth muscles in absence of dystrophin expression in the *mdx* mouse [20–22]. TRPC3 acts as a positive regulator of reactive oxygen species, its increased expression leading to a fibrotic response in cardiomyocytes [20]. TRPC3 has also been proposed to participate in the massive and sustained cytosolic Ca^2+^ increase that takes place in skeletal muscle cells during malignant hyperthermia (MH) with numerous MH like episodes having been reported in human DMD patients [23, 24]. The two channels are involved in myogenesis and regulate cytosolic Ca^2+^ levels in skeletal muscle fibers [25, 26]. Targeting TRPC1 and TRPC3 to reduce Ca^2+^ alterations in DMD muscles could thus represent relevant targets for alternative or complementary treatment to MD-based gene therapy.

Nevertheless, most of the studies concerning the involvement of Ca^2+^ homeostasis alterations and the TRP channels in the pathogenesis of DMD were conducted in the *mdx* mouse. This animal model of DMD exhibits a very mild muscle dystrophy as compared to DMD patients [27, 28]. This reduces the scope of the results obtained in *mdx* mice in the understanding of the disease and the development of treatments in human DMD patients. Our team participated to the generation of the *DMD*^*mdx*^ rat model [27], which more closely reproduces the human DMD disease with progressive and severe skeletal muscle replacement by fibrosis, significant reduction in muscle strength, a decrease in spontaneous motor activity and cardiac involvement.

In the present study, we aimed to determine whether TRPC1 and/or TRPC3 channels may be involved in skeletal muscle SPCa alterations in the *DMD*^*mdx*^ rat and may represent therapeutic targets. We assessed [Ca^2+^]_c_ and SPCa in mechanically isolated and fura-2 loaded fibers of the EDL (*Extensor Digitorum Longus*) fast-twitch muscle from age-match wild-type (WT) and *DMD*^*mdx*^ rats of 1.5–7 months old. Within this time window, rats undergo puberty and reach adulthood [29] and, most importantly, *DMD*^*mdx*^ animals progressively display necrosis and regeneration in limb and diaphragm muscles that evolves to severe fibrosis and adipose tissue infiltration [27]. TRPC1 and TRPC3 expressions were measured in the EDL muscles at both the mRNA and protein levels, by RT-qPCR and western blot analysis, respectively. The subcellular localization of the two channels was assessed by immunocytofluorescence and confocal microscopy. Finally, we determined the impact of a rAAV-MD based treatment on Ca^2+^ homeostasis, force development and TRPC expression in *DMD*^*mdx*^ rat skeletal muscles.

## Materials and methods

### Animals

A total of 69 *DMD*^*mdx*^ rats and 59 Sprague Dawley WT rats (littermates) were used in this study. They were obtained, handled and housed from the UTE IRS-UN (University de Nantes, France) and the Boisbonne Center for Gene Therapy (ONIRIS, Nantes, France). The Institutional Animal Care and Use Committee of the Région des Pays de la Loire (University of Angers, France) as well as the French Ministry for National Education, Higher Education and Research approved the protocol (authorizations #2016070618053653 and 2017040616371353). Before sacrifice, animals received a subcutaneous injection of buprenorphine (0.04 mg/kg, Vetergesic, Ceva Santé Animale, Libourne, France), after 30 min rats were anesthetized by intraperitoneal injection with etomidate (16 mg/kg, Hypnomidate, Janssen-Cilag, Issy Les Moulineaux, France), delivered in 2 or 3 injections separated by 3 min, and ketamine (20 mg/kg, Imalgene 1000, Merial, Lyon, France). Animals dedicated to ex vivo skeletal muscle contractility analysis and Ca^2+^ measurements were euthanized by heart excision. Other animals were euthanized by intravenous injection of pentobarbital sodium (Dolethal, Vetoquinol, Paris, France).

### Histology

Some rats that died prematurely during this study were necropsied. A few tissues and organs (including heart, lung, kidney, liver, spleen, skeletal muscle and brain) were then obtained for immediate fixation in formalin. After paraffin embedding, 4 µm-thick sections were further stained using Hematoxylin–Eosin-Saffran routine protocol. Additional tissue staining (Picrosirius red and von Kossa for Ca^2+^) was performed on skeletal muscle tissues when needed. These tissue samples were observed by a veterinary pathologist to determine the cause of death.

### Ex-vivo skeletal muscle contractility

Isometric contractile properties of the EDL muscles were evaluated according to methods previously described [30]. Briefly, muscles were removed from the hindlimb of anesthetized rats and mounted in an in vitro muscle test system (1205A model; Aurora Scientific, Aurora, Canada). Muscles were placed between two platinum electrodes in a muscle bath containing 100 ml of bubbled mammalian Ringer solution at 25 °C. After a 5 min equilibration period, optimum muscle length was determined by gradual muscle length adjustments and eliciting isometric contractions (supramaximal square-wave pulses of 0.2 ms duration) until the maximum twitch tension was reached. After 5 min of rest, muscles were stimulated at 10, 20, 40, 60, 80, 100, 120 Hz for 500 ms at each frequency. Stimulus trains were separated by 1-min intervals. Maximum isometric tetanic force was determined from the plateau of this force frequency curve. Following force testing, muscles were removed from the bath, trimmed of tendons, and weighed. Muscle mass was then be used to calculate maximum tetanic specific force in g/g.

### Dissection of native muscle fibers

For in vitro experiments, EDL muscles were removed from the animal under deep anesthesia and were pinned in a dissecting dish containing normal physiological solution (NPS) at room temperature (22 °C) for further dissection. NPS contained the following: 140 mM NaCl, 5 mM KCl, 1 mM MgCl_2_ (all from VWR International, Fontenay sous Bois, France), 10 mM HEPES, 10 mM glucose, and 1.8 mM CaCl_2_ (all from Sigma-Aldrich, Saint Quentin Fallavier, France) at pH 7.35. Contralateral EDL muscles of some animals were snap-frozen and stored at − 80 °C for biochemical and molecular biology as described below. Skeletal muscle fibers from EDL muscle of the different groups of rats were dissected intact. Small bundles of 10–15 fibers arranged in a single layer were dissected lengthwise, tendon to tendon, with the use of microscissors, as described elsewhere [31]. Part of the bundles were used for Ca^2+^ measurements and the remainder kept for immunofluorescence experiments.

### Cytosolic Ca^2+^ and sarcolemmal permeability to divalent cation measurements

EDL muscle bundles were incubated in NPS containing 5 µmol/l Fura-2 AM (Molecular Probes, OR, USA) for 1 h at room temperature, rinsed twice, and left 30 min before use to ensure complete de-esterification. Ratiometric Fura-2 fluorescence measurements were made using an integrated IonOptix (IonOptix, Amsterdam, Netherlands) device and excitation filters of 360 and 380 nm. Emitted fluorescence (510 nm) was background subtracted. The cytosolic Ca^2+^ concentration ([Ca^2+^]_c_) was calculated from ratiometric measurements according to a modified method from Grynkiewicz and colleagues [31, 32].

The manganese quenching technique was used to determine the sarcolemmal permeability to divalent cations (SPCa). Muscle preparations were first perfused for 2 min with NPS containing 0.5 mM Mn^2+^ as a surrogate of Ca^2+^ (quenching solution). Then, the quenching solution was applied to muscle fibers for 2–4 min. During the whole quenching protocol, the fluorescence of Fura-2 excited at 360 nm was acquired at 1 Hz. The quench rates were determined using linear regression analysis of fluorescence signal and expressed as the decline per minute of the initial fluorescence intensity. For experiments dedicated to evaluate Pyr10 (–N-(4-(3,5-bis(trifluoromethyl)-1H-pyrazole-1-yl)phenyl)-4- methylbenzenesulfonamide), quench rate was first measured in quenching solution. Muscle fibers were washed and then incubated 10 min in NPS without Ca^2+^ but containing 3 µM of Pyr10 [33]. Quench rate was then newly measured in previous fibers in quenching solution containing 3 µM Pyr10.

### TRPC1 and TRPC3 submembrane distribution analysis by immunofluorescence

EDL muscle fiber bundles containing 5 to 10 fibers were dissected with microscissors in skinning solution and were immunolabelled according to a method adapted from Liu and colleagues [34]. Briefly, muscle fiber bundles were stretched in Sylgard 184 silicone elastomer (Sigma, France) molded chambers designed to maintain the muscle fibers at tension allowing to obtain a sarcomere length close to that found in vivo (~ 2.5 µm) during the whole immunolabelling process. Muscle bundles were then fixed using PBS containing 4% PFA (ThermoFisher, Massachusetts, USA) for 1 h, then permeabilized using 2% TritonX 100 diluted in PBS for another hour under agitation. After washing, muscle bundles were incubated during 48 h under agitation in a PBS solution containing goat polyclonal anti-caveoline-3 (1:50, Bio-Techne, Minnesota, USA) and mouse anti-ryanodine receptor (1:100, DSHB, Iowa, USA) antibodies. Donkey serum was added at 5% in this latter solution. After washing muscle bundles were newly incubated for 48 h under agitation in a PBS solution containing 5% of donkey serum, donkey anti-goat Alexa Fluor 647 conjugated (1:400) and donkey anti-mouse cyanine-3 conjugated (1:400) (Jackson ImmunoResearch, UK). After a new wash, muscle fibers were newly fixed using 4% PFA PBS for 15 min. Finally, muscle fibers were incubated in a PBS solution containing mouse anti-TRPC1 or anti-TRPC3 antibodies Alexa Fluor 488 conjugated (1:50, Santa Cruz Biotechnology, Texas, USA) for 48 h under agitation. After final wash, muscle preparations were mounted in ProlonGold (ThermoFisher, Massachusettes, USA) between glass cover slip and slide. Image Z-stacks were acquired using a Nikon confocal A1 N-SIM microscope with a Plan Apo X60 objective (Nikon France Sas, Champigny-Marne, France).

Images of the acquired stack corresponding to the center of muscle fibers were used for analysis. A macro was written under ImageJ macro language to routinely and semi-automatically analyze the images. Briefly, the experimenter was asked to enter the number of the cells to analyze in a rectangle Region Of Interest (ROI) and to delimitate the peaks corresponding to Cav-3 labelling. A maximum peak was then automatically calculated and defined as sarcolemma reference position. This position was then used to calculate into the stack, for each channel corresponding to TRPC1 or TRPC3 labeling, the areas of the fiber corresponding to the 5 µm spaces beneath sarcolemma and the cell center, and their corresponding integrated signal density.

### Relative quantification of TRPC1, TRPC3 and MD messengers by RT-qPCR

Total RNA was extracted from pieces of EDL muscles from the different groups of rats with QIAzol Lysis Reagent (Qiagen, Germany) according to the manufacturer’s instructions. Then, 1000 ng of total RNA was treated with RNAse-free DNAse I (ezDNAse from ThermoFisher, Massachusetts, USA) and reverse transcribed using SuperScript IV Vilo reverse transcriptase and random primers (ThermoFisher, Massachusetts, USA) in a final volume of 20 µL. qPCR analysis was then performed on cDNA (diluted 1/40 for TRPC1 and 1/80 for TRPC3) using different primers designed to amplify a specific region of the TRPC1 messenger (Forward: TTCCAAAGAGCAGAAGGACTG and Reverse: AGGTGCCAATGAACGAGTG according to Sabourin and collaborators [35]), the TRPC3 messenger (Forward: ACGCTTCTCACCTGACATCA and Reverse: CTGGACAGCGACAAGTATGC) or the MD messenger (Forward: CCAACAAAGTGCCCTACTACATC, Reverse: GGTTGTGCTGGTCCAGGGCGT, and Probe: CCGAGCTGTACCAGAGCCTGGCC). As an internal control, HPRT1 messenger was used to normalize the mRNA concentration (Forward: GCGAAAGTGGAAAAGCCAAGT, Reverse: GCCACATCAACAGGACTCTTGTAG, Probe: CAAAGCCTAAAAGACAGCGGCAAGTTGAAT). Results were expressed in relative quantities (RQ): RQ = 2^−∆Ct^ = 2^−(Ct target−Ct endogenous control)^. For each RNA sample, the absence of DNA contamination was also confirmed by analysis of “cDNA liked samples” obtained without addition of reverse transcriptase in the reaction mix.

### rTRPC3 cDNA amplification and sequencing

PCR amplification of the coding region around the exon 9 of the TRPC3 cDNA was performed on total cDNA from EDL of WT and *DMD*^*mdx*^ rats. PCR was performed using LA Taq polymerase (Takara, Kusatsu, Japan) and the primers published by Kim and collaborators [36]: Forward: CAGTGATGTAGAGTGGAAGTTTGC, Reverse: CTCCCTCATTCACACCTCAGC. The amplification products were loaded on a 2% agarose gel. The amplification of the full-size cDNA of TRPC3 was performed using the following primers: Forward: ACGCAGTACGGCAACATCC, and Reverse: CATTCACACCTCAGCGCACT. The amplification products were then sequenced with Sanger method (Genewiz, South Plainfield, USA).

### Western blot analysis of TRPC1 and TRPC3 expression

In order to extract EDL muscle total proteins, muscles were homogenized using TissueLyser II (Qiagen, Germany) in RIPA buffer containing a protease inhibitor cocktail (Sigma-Aldrich, Missouri, USA). 50 µg of protein extracts, denatured 10 min at 70 °C with Laemmli (Biorad, California, USA), were loaded on a 10% Tris–Glycine Precast polyacrylamide gels (ThermoFisher, Missouri, USA). After 2 h of migration at 100 V, and Red Ponceau staining, membranes were blocked over-night at 4 °C (PBS-Tween 0.1%, non-fat dry milk 5% and NP40 1%). Then, membranes were incubated 1 h at room temperature with mouse anti-TRPC1 antibody (1:50 000, sc-133076; Sant Cruz Biotechnology, Texas, USA), mouse anti-TRPC3 (1:500, sc-514670; Sant Cruz Biotechnology, Texas, USA) or goat anti-GAPDH (1:10,000, Novus Biologicals, Colorado, USA). After washing with PBS Tween 0.1% membranes were incubated with secondary rabbit anti-mouse HRP (1:5000) or rabbit anti-goat HRP (1:2000; Agilent Technology, California, USA). After washing with PBS Tween 0.1%, ECL (ThermoFisher, Massachusetts, USA) was applied on membranes and films (Amersham Hyperfilm™) were exposed to it. MD protein expression in EDL was analyzed as previously published [11]. Relative protein expressions were calculated by normalizing signal intensity measured using ImageJ software by GAPDH signal of the corresponding lane and the signal of an experimental sample that was loaded on every gels (ImageJ). This latter allowed us the comparison between gels.

### TRPC3 post-translational analysis

Deglycosylation analysis of the TRPC 3 protein was performed using Protein Deglycosylation Mix II kit (New England Biolabs, Ipswich, USA). Briefly, 40 µg of total protein extract were diluted in water. Then, 2 µL of Deglycosylation Mix Buffer 2 were added to the proteins and the mix was incubated at 75 °C for 10 min. Next, 2 µL of the enzyme Protein Deglycosylation Mix II were added to the proteins, followed by incubations 30 min at room temperature and 1 h at 37 °C. Positive control (Fetuin) was provided in the kit. The results were analyzed by western blot as described previously. Dephosphorylation analysis of the TRPC3 protein was performed using Fast AP Thermosensitive Alkaline Phosphatase kit (ThermoFisher, Massachusetts, USA). The reaction was performed on 30 µg of total protein extract diluted in water. Briefly, a mix of 8 µL of 10X FastAP buffer and 60 µL of FastAP Phosphatase were added to proteins. The reaction mix was incubated 1 h at 37 °C. The proteins were then concentrated by adding 320 µL of acetone to the reaction mix, followed by an overnight incubation at − 20 °C, and a centrifugation at 13,000 rpm during 15 min at 4 °C. The pellet was then resuspended in water, and analyzed by western blot as described above.

In order to accurately calculate the apparent molecular weight (AMW) and allows comparison between lanes a dedicated macro was built under ImageJ and dedicated for selected western blots. In each gel four experimental samples were flanled by two ladder samples to allow robust size determination and limit separation artefacts. The ImageJ macro was designed to automatically determine the relation between distance of migration and ladder sizes surrounding the size of interest according to a Botlzmann curve fit. The parameters of the Boltzmann equation were then used to calculate the AMW protein of interest starting from distance migration.

### rAAV-MD injection in *DMD*^*mdx*^ rats

Murine-specific cDNA sequences of optimized MD version 1 have been previously described [37, 38]. This MD cDNA is deleted of spectrin-like repeat domain 4–23 and CT domain (exons 71–78) and contains the last three amino acids of exon 79 of dystrophin followed by three stop codons [37]. MD cDNA sequence was subcloned into a pAAV plasmid that contained the 323 bp muscle-synthetic Spc5.12 promoter [39], a synthetic polyadenylation signal of 49 bp obtained from the pCI-neo plasmid (Promega, Madison, WI), and two flanking inverted terminal repeat (ITR) sequences of 130 pb from AAV serotype 2. The size of the resulting MD expression cassette (including Spc5.12 promoter, pCI-Neo polyA, and ITRs) was 4538 bp. Recombinant pseudo typed AAV2/9-MD vectors were produced by the Vector Core of the UMR 1089 (CPV, INSERM and University of Nantes) by transient transfection of HEK293 cells followed by purification on cesium chloride density gradients. Final vectors were concentrated and formulated in Dulbecco's phosphate-buffered saline (DPBS, Fisher Scientific, Illkirsch, France), sterile filtered, aliquoted and frozen at ≤ − 70 °C. Vector genome titers (vg/mL) were determined using a qPCR assay specific for ITR264 [40].

Prior to injection, the rAAV vectors were diluted in Dulbecco’s phosphate-buffered saline (DPBS) vehicle solution to obtain a fixed total volume corresponding to 15 mL of perfusate per kg of animal. Injections were performed without anesthesia but under analgesic premedication, performed at least 30 min before injection by subcutaneous injection of buprenorphine at 0.04 mg/kg. Vector or its vehicle was administered at the age of 1 month by the intravenous route in a tail vein at a fixed flow rate of 0.5 mL/min. Animals were sacrificed after 3 months of follow-up.

### Diaphragm ultrasonography in vivo

The technique was adapted from Whitehead and colleagues [41]. Briefly, ultrasonography was performed using a Vivid 7 ultrasound unit (GE Healthcare, Velizy Villacoublay, France) associated to a 14 MHz M12L probe. First, animals received a subcutaneous injection of buprenorphine, after 30 min rats were anesthetized by intraperitoneal injection with etomidate (16 mg/kg, Hypnomidate, Janssen-Cilag, Issy Les Moulineaux, France), the hair on the chest and abdomen was removed using hair-removal cream. Once anaesthetized, the rat was placed supine on the imaging platform and the four limbs. The platform was pre-heated to maintain the core body temperature at 37 °C, which was monitored with a temperature-sensitive rectal probe. Ultrasound gel was applied to the area overlying the diaphragm and liver. The probe was manually positioned 120° relative to the rat platform. The probe was placed along the transverse mid-sternal axis of the rat, in order to locate the diaphragm on both sides of the body. According to Whitehead and colleagues in the mouse the liver and portal vessels were used as landmarks [41]. M-mode was used to measure the diaphragm movement during normal breathing cycles. The M-mode image window was positioned on the left side of the sternum, over a flat region of diaphragm. Images were then recorded during at least 15 breathing cycles. In order to avoid experimenter and probe positioning artifacts, the probe was removed and replaced 2 times to allow acquisition of 3 records. A semi-automatic analysis method was then applied to measure the amplitude of the diaphragm movement during each inspiration. Each recorded image was filtered using ImageJ (Gaussian Blur > Threshold > Find Edges) and transformed in (x, y) calibrated curves using GetData Graph Digitizer 2.26. A macro was built under ImageJ to automatically calculate the amplitude of diaphragm contraction (the difference in mm between baseline and the peak of the contraction) of 5 consecutive cycles.

### Statistics

All statistical analyses were performed using XLStat software (Addinsoft, Paris, France). P-values < 0.05 were considered statistically significant. The statistical tests were specified in the text and were choose depending on data number in each group and the number of groups to be tested for a given parameter.

## Results

### Malignant hyperthermia episode

**Fig. 1:**
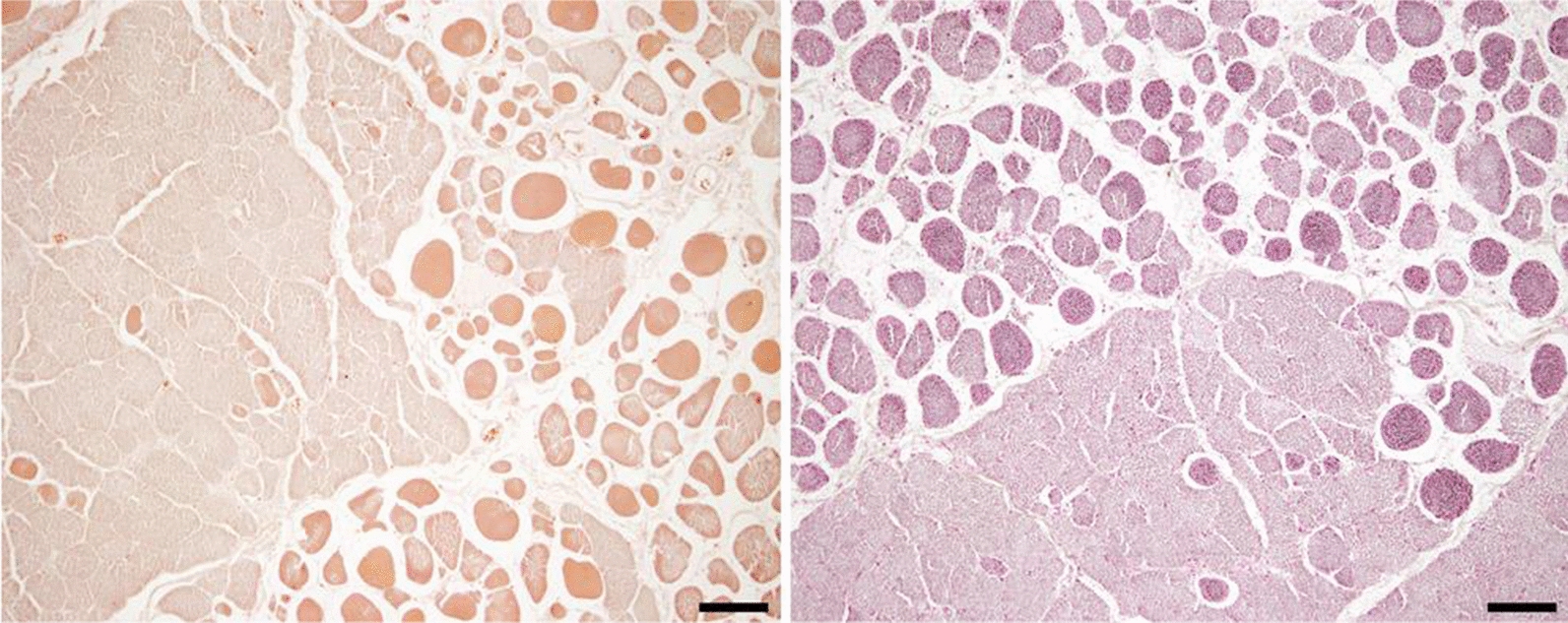
Histopathological evaluation of skeletal muscles from *DMD*^*mdx*^ rats deceased following malignant hyperthermia syndrome. After specific staining for calcium (left panel: alizarin red and right panel: von Kossa), we observed differences of dye affinity between normal fibers and round hypercontracted fibers that highlighted disruption of calcium concentrations between intracellular and extracellular compartments. Note the severe endomysial edema typical of hyperthermia syndrome. Bar = 100 µm

At the beginning of this study, some few *DMD*^*mdx*^ rats were anesthetized using a halogenated agent (isoflurane) in order to obtain control blood samples. Unexpectedly, 3 out of 16 rats died during the course of anesthesia. These rats were necropsied and main organs and tissues were analyzed by a pathologist to determine the cause of death. All these rats typically exhibited similar myopathic lesions typical of the model [27]. These lesions included isolated hyalin fibers, small clusters of degenerative fibers associated with muscle fiber regeneration foci, centro-nucleated fibers and anisocytosis with a few inflammatory cells in a slightly increased endomysial space corresponding to mild fibrosis. In addition to these classical DMD lesions, these rats displayed (1) large clusters of round hypereosinophilic fibers (hypercontracted fibers) corresponding globally to half of the total number of fibers. Some of them displayed fragmented cytoplasm and/or hyperchromatic condensed or fragmented nucleus indicative of necrosis (severe rhabdomyolysis), (2) huge optically empty space between fibers corresponding to massive edema. Using Picrosirius and von Kossa stainings, both specific for Ca^2+^, an increased concentration of Ca^2+^ was identified in hypercontracted fibers (Fig. 1). The association of these lesions are typical of malignant hyperthermia (MH) or MH-like reaction. This syndrome is responsible for the immediate death of the animal notably due to neuronal death associated to severe hyperthermia, as evidenced in some of these animals in which brain tissue conservation was good. Interestingly, similar findings were observed in 5 rats of another study that were found dead during or immediately after an experiment requiring long-lasting posture constraint. As developed in the discussion, these MH events were first clues indicating muscle Ca^2+^ homeostasis alterations in the *DMD*^*mdx*^ rats.

### Ca^2+^ homeostasis is dysregulated in* DMD*^*mdx*^ EDL muscles

**Fig. 2:**
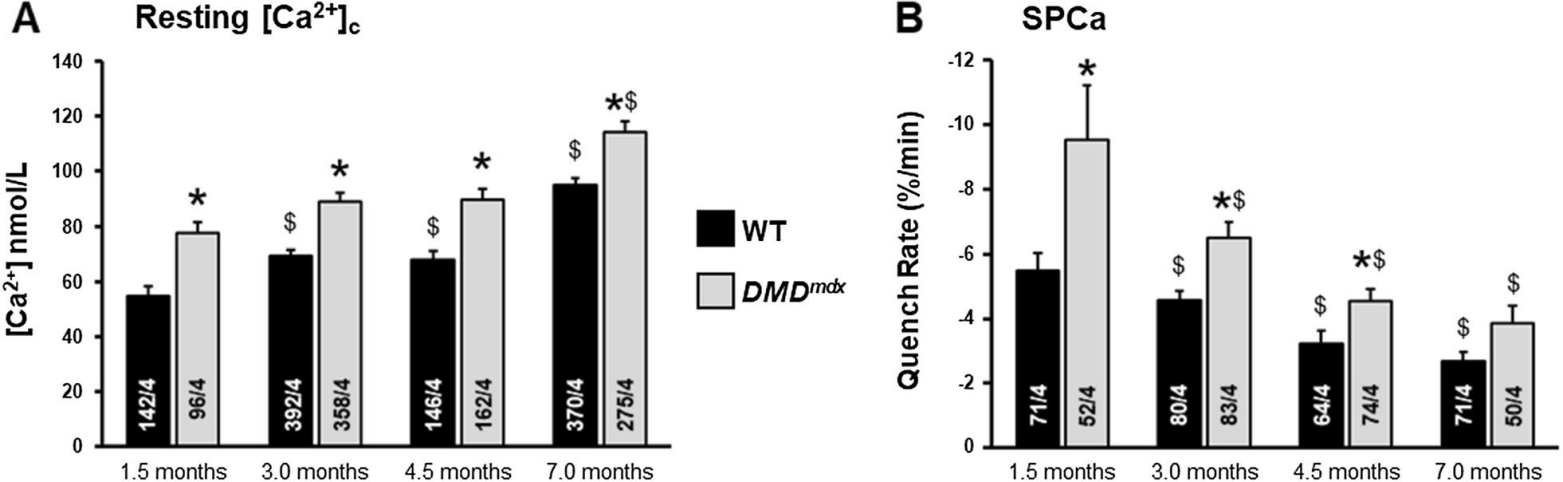
Evolution of resting calcium homeostasis in EDL muscle fibers from WT and *DMD*^*mdx*^ rats during post-natal development. **A** Resting cytosolic calcium concentration ([Ca^2+^]_c_) **B** Resting sarcolemmal permeability to calcium (SPCa). [Ca^2+^]_c_ and SPCa were measured in Fura2 loaded single fibers from mechanically isolated bundles of EDL muscles in WT and *DMD*^*mdx*^ rats of 1.5 to 7.0 months of age. SPCa was measured using the manganese quenching of Fura2 technique. Each bar corresponds to mean value ± SEM measured in n single fibers from N animals (n/N are indicated at the bottom the bars). *: significantly different from mean value measured in age-matched WT animals. $: significantly different from mean value measured in animals from the same genotype of 1.5 months of age. P < 0.05, Two-way ANOVA and Fisher-LSD post-hoc test for pairwise comparisons

From the age of 1.5 to 7 months, resting [Ca^2+^]_c_ and SPCa were determined in each single fura-2-loaded fiber constituting the bundles dissected from the EDL muscles of WT and *DMD*^*mdx*^ rats. During this period, resting [Ca^2+^]_c_ levels showed slight but significant increase in WT EDL muscle fibers (Fig. 2A). A similar increase was observed in *DMD*^*mdx*^ muscles, but at each time-point the mean [Ca^2+^]_c_ was ~ 20 to 30% significantly higher than in WT muscle fibers. In the same muscle bundles, we used the Mn^*2*+^ quenching technique in order to determine whether resting [Ca^2+^]_c_ rise was associated with an increase in the sarcolemmal permeability to Ca^2+^ (SPCa). In contrast with [Ca^2+^]_c_, SPCa progressively and significantly decreased in muscle fibers from WT rats from 1.5 to 7 months of age (Fig. 2B). Age-dependent decrease of SPCa was also observed in *DMD*^*mdx*^ muscle fibers but the mean values were always significantly higher in dystrophic fibers than in age-matched WT controls. This difference was particularly high at 1.5 months of age (~ 50%) and progressively decreased to reach ~ 35% at 7 months. In order to assess whether the elevation of [Ca^2+^]_c_ and SPCa in *DMD*^*mdx*^ EDL were associated with changes in TRPC1 and/or TRPC3 expression we conducted a series of experiments to measure mRNA and protein levels of the two channels.

### Expression of TRPC1 and TRPC3 channels are modified over the course of the DMD pathology in* DMD*^*mdx*^ rats

**Fig. 3:**
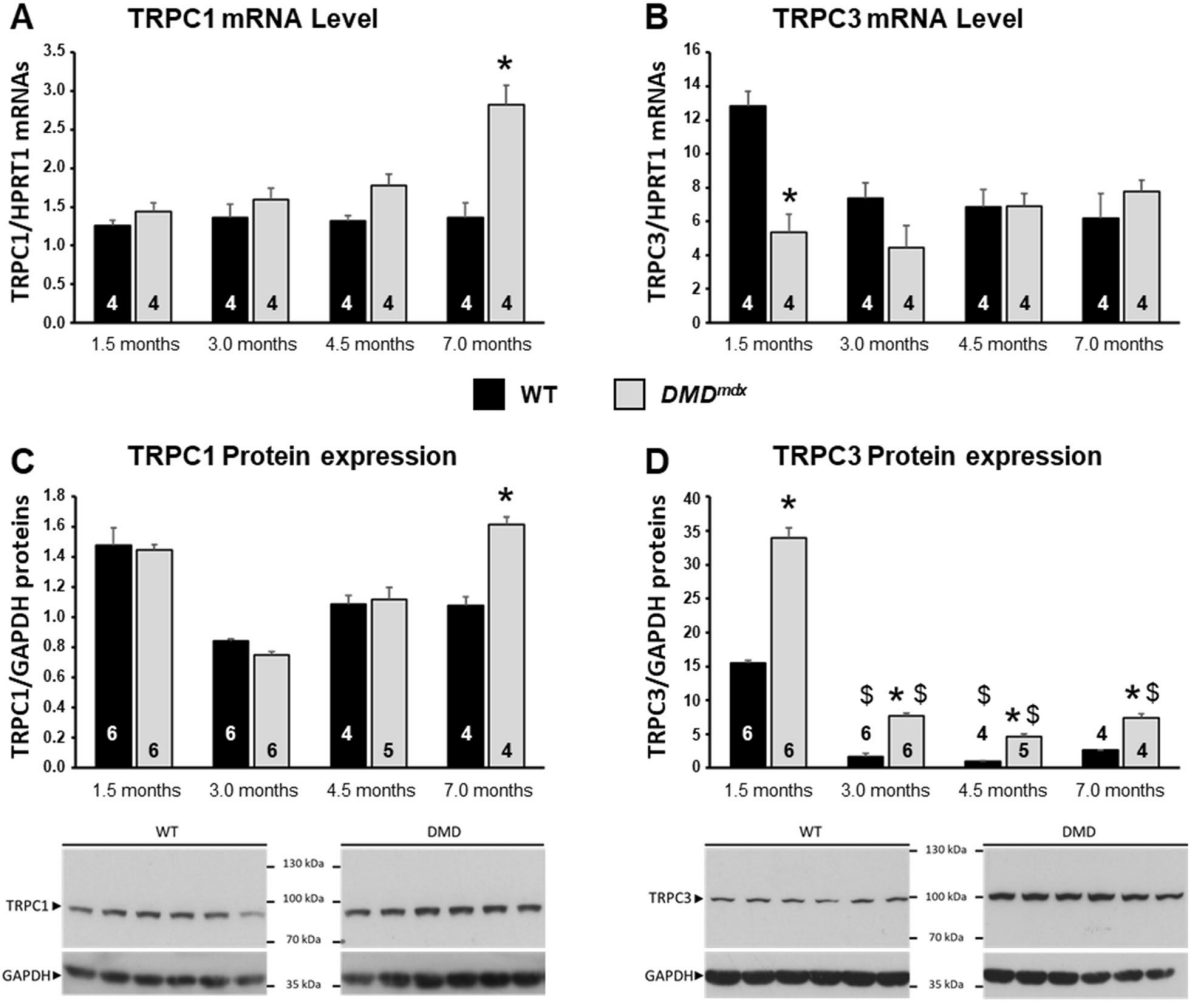
Evolution of TRPC1 and TRPC3 mRNA and protein expression levels in EDL muscles from WT and *DMD*^*mdx*^ rats during post-natal development. **A and B** Relative levels of TRPC1 and TRPC3 mRNAs expression in EDL muscle extracts from WT and *DMD*^*mdx*^ rats of 1.5 to 7 months of age. Results were normalized to HPRT1 mRNA expression. **C and D** Relative levels of protein expression of TRPC1 and TRPC3 in EDL muscle homogenates from WT and *DMD*^*mdx*^ rats of 1.5–7 months of age. Representative films exposed to immunoblots are reported below histograms (obtained for muscle extracts from 6 WT and 6 *DMD*^*mdx*^ rats of 1.5 months of age). **A–D** Each bar represents mean value ± SEM calculated for N animals (N indicated at the bottom of the corresponding bar). For each age and genotype, immunoblots were realized in a minimum of 3 replicates. *: significantly different from mean value measured in age-matched WT animals. $: significantly different from mean value measured in animals from the same genotype of 1.5 months of age. P < 0.05, Kruskal–Wallis and bilateral Conover-Iman post-hoc test

Muscle homogenates from WT and *DMD*^*mdx*^ rats of 1.5–7 months of age were analyzed by RT-qPCR and western blot (Fig. 3). In WT rats, TRPC1 mRNA expression level was stable all over ages tested (Fig. 3A). TRPC1 protein expression level was heterogeneous among ages with a tendency at overexpression at 1.5 months of age, but without statistical significance (Fig. 3C). Similar results were obtained for *DMD*^*mdx*^ rats except at 7 months of age, where TRPC1 mRNA and protein expression levels were significantly higher than in WT controls (Fig. 3A–C). Results were quite different for TRPC3 (Fig. 3B–D). In WT EDL muscles, both mRNA and protein expression levels were the highest at 1.5 months of age and significantly decreased thereafter to reach a steady-state level at 3 months of age. In *DMD*^*mdx*^ EDL muscles, TRPC3 mRNA expression levels were significantly lower to that of WT ones at 1.5 months of age. From 3 to 7 months of age, TRPC3 mRNA expression level was stable and similar to that measured in WT animals. In contrast to mRNA, TRPC3 protein expression level was ~ twofold higher in *DMD*^*mdx*^ than in WT rats at 1.5 months of age (Fig. 3D). Although TRPC3 protein expression decreased with age in both *DMD*^*mdx*^ and WT rats, it always remained ~ twofold higher in dystrophic animals. It is notable that TRPC translocation from intracellular membrane compartments (e.g., sarcoplasmic reticulum) to the sarcolemma could lead to an increase in SPCa. This prompted us to examine the TRPC subcellular localization in muscle.

### Subcellular localization of TRPC1 and TRPC3 is not modified in* DMD*^*mdx*^ muscles

**Fig. 4:**
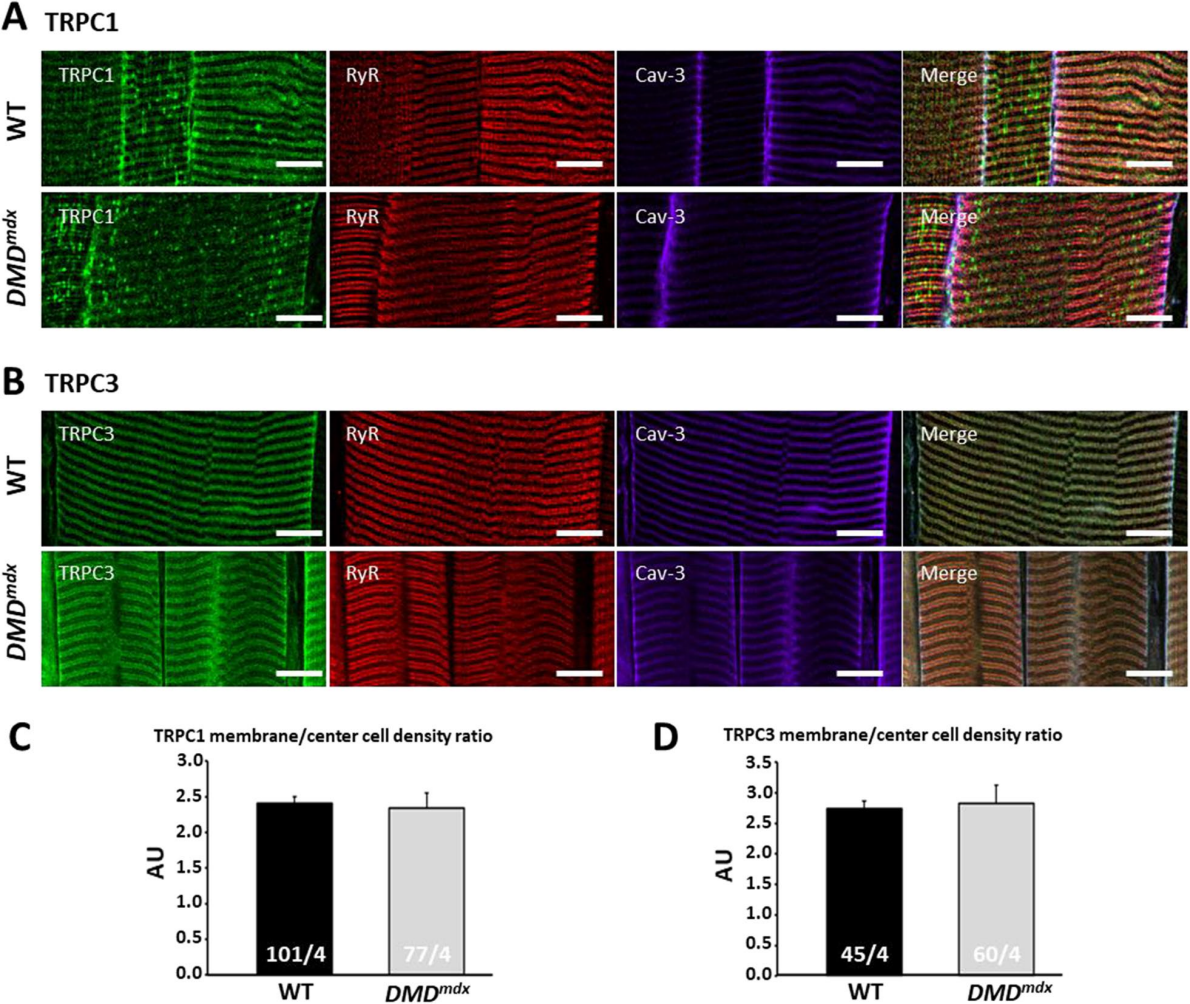
Subcellular localization of TRPC1 and TRPC3 protein expression in EDL muscle fibers from WT and *DMD*^*mdx*^ rats. **A and B** Confocal images acquired in longitudinal axis and at the largest diameter of EDL muscle fibers from WT and *DMD*^*mdx*^ rats of 4.5 months of age. Fibers were fixed, permeabilized and co-labeled by immunofluorescence to highlight TRPC1 or TRPC3 protein expression and compare it to RyR and Cav-3 ones. A and B are montages of pictures cropped from original images acquired using a X60 objective. Scale bar are reported in white and corresponds to 14 µm. **C and D** TRPC1 and TRPC3, respectively, sub-peripheral sarcolemma area (peripheral sarcolemma labeled using Cav-3 antibodies and 5 µm beneath area) to fiber center (the rest of the intracellular area) density signal ratio. Each bar represents mean ± SEM value of density ratios measured in n muscle EDL muscle fibers and N animals (n/N are noted at the bottom of each bar). Potential significant differences were evaluated using Student’s unpaired *t*-test

We investigated the subcellular localization of TRPC1 and TRPC3 proteins in WT and *DMD*^*mdx*^ muscle fibers by immunofluorescence and confocal microscopy to determine the proportion of TRPC channels expressed at the peripheral sarcolemma compared to the center of the cells. Caveoline-3 (Cav-3) and the Ryanodine Receptors (RyR) were used as sarcolemma and sarcoplasmic reticulum markers, respectively (Fig. 4A, B). Despite a heterogeneous and partly punctiform location, TRPC1 was mostly observed at the peripheral sarcolemma where it seemed to colocalize with Cav-3 (Fig. 4A). Similar sarcolemmal location was seen for TRPC3 with a more homogeneous labelling (Fig. 4B). In the center of the cells, the immunofluorescence signals for both channels appeared in a typical striated pattern. Comparison of Cav-3 and RyR immunolabeling to that of TRPC1 and TRPC3 did not allow us to clearly determine the cellular membrane compartments where TRPCs were expressed. This may be due to the high entanglement of t-tubules and sarcoplasmic reticulum (SR) terminal cisternae that form highly specialized ultrastructure in muscle fiber cells, called triads [42]. In order to further analyze the potential difference in location between WT and *DMD*^*mdx*^ muscle fibers, the density signal ratios of TRPC1 and TRPC3 between the peripheral sarcolemma and the center of the cells were calculated (Fig. 4C, D, see “Material and methods” section for details). No significant difference was observed between dystrophic and healthy muscle fibers. Considering the late expression alteration of TRPC1 and the earliest one for TRPC3, and that no significant change in location change is observed for the two channels, we focused our attention on TRPC3.

### Post-translational TRPC3 modification changes in *DMD*^*mdx*^ rat skeletal muscle

**Fig. 5:**
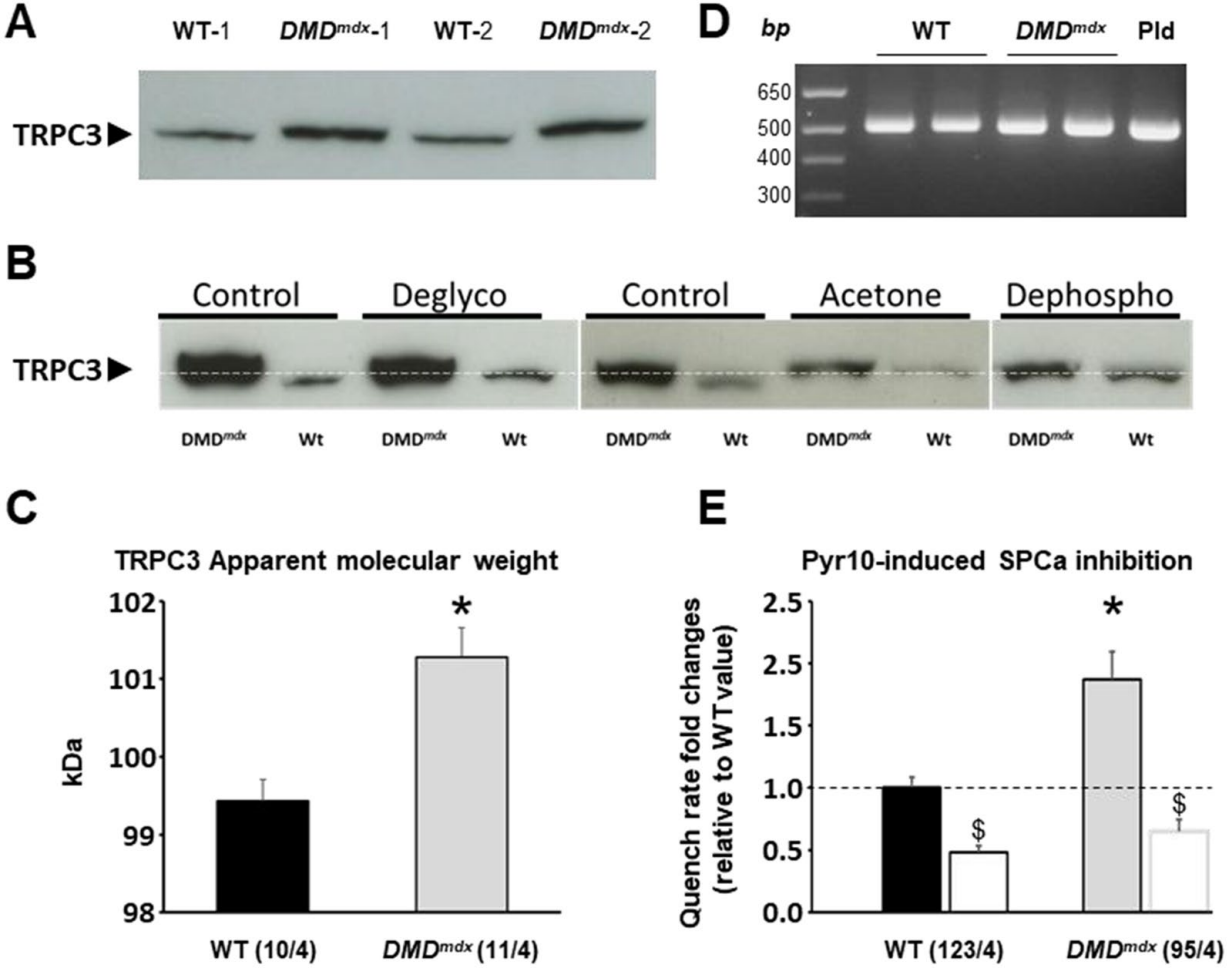
TRPC3 apparent molecular weight (AMW) and Pyr10-induced SPCa inhibition change between WT and *DMD*^*mdx*^ rats in EDL muscle. **A** Immunoblot of TRPC3 of EDL muscle homogenates from 4 different rats (2 WT: WT-1 and WT-2 and 2 *DMD*^*mdx*^: *DMD*^*mdx*^-1 and *DMD*^*mdx*^-2) of 4.5 months of age. WT and *DMD*^*mdx*^ homogenates were alternated in the different lanes to underline the difference in AMW. **B** Relation between glycosylation and phosphorylation status and AMW. Immunoblots of TRPC3 of EDL muscle from the same *DMD*^*mdx*^ and WT rats in Control conditions (Control) or after deglycosylation (Deglyco), acetone treatment only (Acetone) and, acetone treatment and dephosphorylation (Dephospho). **C** AMW of TRPC3 measured in immunoblots for EDL muscle homogenates from 4 WT and 4 *DMD*^*mdx*^ rats. Measures were made for at least two replicates for each rat. Bars represent mean ± values. *: significantly different from WT value, Mann–Whitney test, P < 0.05. **D** Agarose gel electrophoresis showing RT-PCR amplicons corresponding to the exon 8 to exon 10 of TRPC3 mRNA from EDL muscles of WT and *DMD*^*mdx*^ rats, and pld as a plasmid expressing the full-length rat TRPC3 mRNA isoform. *bp:* size in base pairs. **E:** Pyr10-induced SPCa inhibition expressed as fold change of the mean value of SPCa measured in control conditions in WT EDL muscle fibers. Bars represent mean ± values of SPCa measured in n EDL muscle fibers from N WT and *DMD*^*mdx*^ rats aged of 4.5 months (n/N are indicated under brackets) before (filled bars) and after (empty bars) application of Pyr10 compound. *: significantly different from WT value measured in the same conditions, Two-way ANOVA and Fisher post-hoc test, P < 0.05. $: significantly different from mean SPCa value measured in control conditions in the same genotype, Paired Student’s *t-*test, P < 0.05

TRPC3 protein expression was increased in *DMD*^*mdx*^ muscles whereas the mRNA expression level was rather decreased as compared to WT. This apparent discrepancy may rely on post-translational modifications altering TRPC3 protein turn-over. As shown in Fig. 5A, we observed a slight but reliable increase in the apparent molecular weight (AMW) of TRPC3 bands revealed on the western blot for *DMD*^*mdx*^ rats. This was observed at the four ages tested (data not shown). We performed additional western blots specifically designed to measure the AMW. Two consecutive lanes containing WT and *DMD*^*mdx*^ EDL muscle homogenates were surrounded by lanes in which a pre-stained protein standard was loaded. After blotting and revelation the AMW of the bands was determined using scanned blots and a homemade ImageJ macro. As shown in Fig. 5C, we found that TRPC3 AMW was ~ 2 kDa higher in *DMD*^*mdx*^ EDL muscles than in WT control muscles. This may be the result of different mRNA transcript variants generated by alternative splicing, or of post-transcriptional modifications. Interestingly, Kim and colleagues reported the alternative splicing of the TRPC3 mRNA exon 9 in the brain of mice, rats, and guinea pigs [36]. They showed that the resulting protein was around 3 kDa smaller than the full-length protein. We compared the size of the amplicons corresponding to a region surrounding TRPC3 exon 9 by RT-PCR (Fig. 5D). There was no significant difference between the amplicons of the two genotypes. We also amplified and sequenced the whole TRPC3 cDNA obtained from EDL, and saw no difference between *DMD*^*mdx*^ and WT rats (data not shown), suggesting that no differential splicing of the TRPC3 mRNA occurs in *DMD*^*mdx*^ rats. Beside mRNA alternative splicing TRPC3 AMW differences between WT and *DMD*^*mdx*^ rats may be due to post-transcriptional modifications. It has been reported that TRPC3 presents one N-glycosylation and several phosphorylation sites [43]. After enzymatic N-deglycosylation treatment, no significant change in TRPC3 AMW was observed in EDL muscle homogenates from *DMD*^*mdx*^ rats. However, in these conditions, TRPC3 AMW in WT rats was shifted towards higher molecular weight values, such thta the difference in TRPC3 AMW between the two genotypes was no longer observed (Fig. 5B). We also assessed the possibility that TRPC3 may be differentially phosphorylated in WT and *DMD*^*mdx*^ muscles. As the experimental kit we used needed high protein concentration, we used the acetone method to concentrate our muscle homogenate. This treatment led to a similar TRPC3 shift in WT muscle homogenates as observed after deglycosylation treatment. The dephosphorylation treatment per se did not further modified TRPC3 AMW (Fig. 5B).

### SPCa inhibition by Pyr10, a TRPC3 specific inhibitor

In order to further assess the possibility that TRPC3 was involved in the SPCa increased in the *DMD*^*mdx*^ EDL muscle fibers, Pyr10, a specific inhibitor of the channel was applied during SPCa measurements (Fig. 5D). For both WT and *DMD*^*mdx*^ application of NPS containing 3 µM of Pyr10 led to a significant inhibition of SPCa. This was more pronounced in dystrophic fibers though the residual mean SPCa values measured in WT and *DMD*^*mdx*^ EDL muscle fibers were not significantly different.

### rAAV microdystrophin gene transfer leads to benefits in Ca^2+^ alterations, TRPC3 expression and skeletal muscle force

**Fig. 6:**
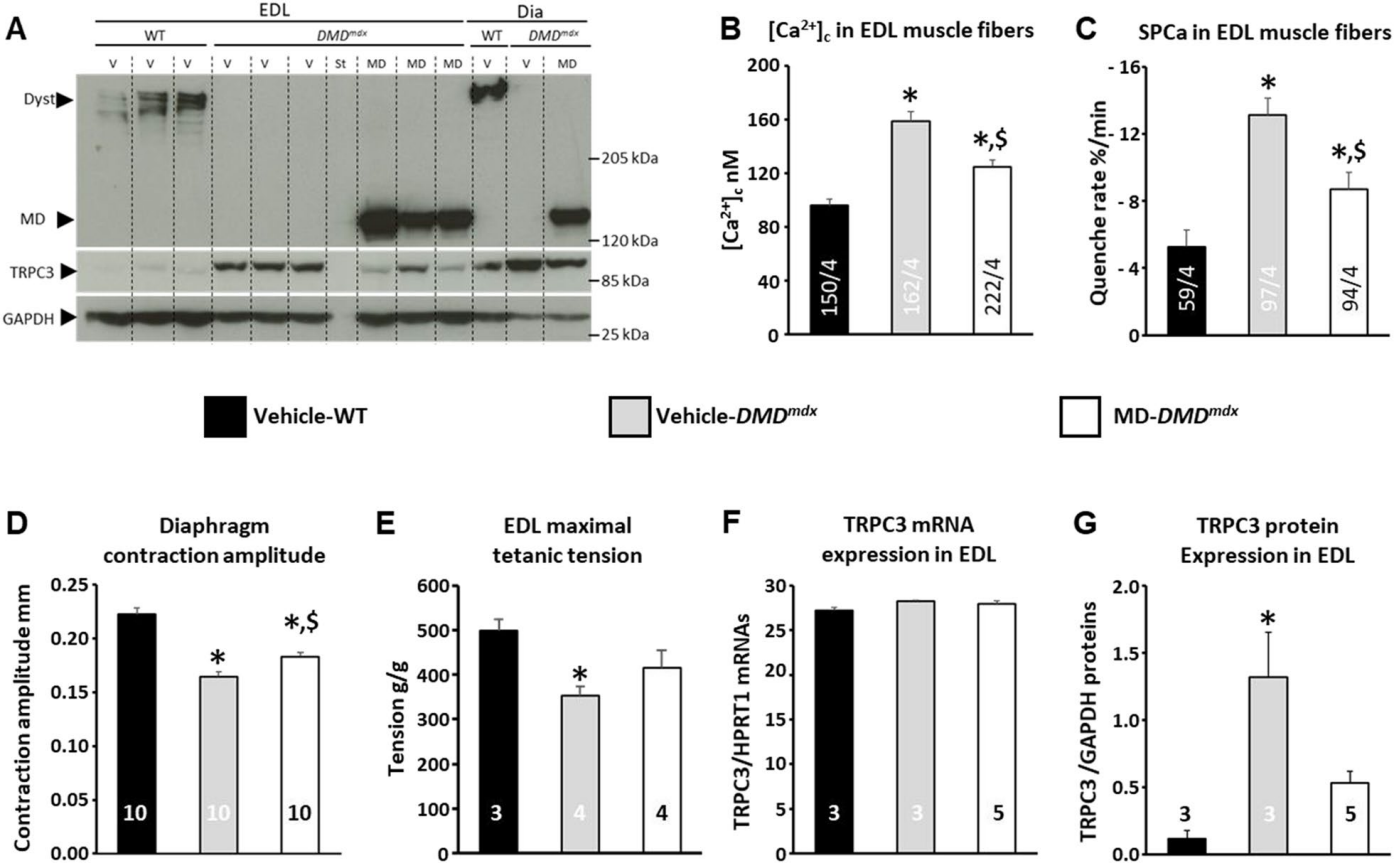
Alterations of calcium homeostasis, TRCP3 mRNA and protein expression levels, and skeletal muscle contraction before and after AAV2/9-MD treatment in *DMD*^*mdx*^ rats. **A** Typical immunoblot labeled to reveal WT dystrophin (Dyst), MD, TRPC3 and GAPDH in EDL and diaphragm (Dia) muscle homogenates obtained from Vehicle-WT, Vehicle-*DMD*^*mdx*^ and MD-*DMD*^*mdx*^ rats. **B** Resting cytosolic calcium concentration ([Ca^2+^]_c_) and **C** Resting sarcolemmal permeability to calcium (SPCa). [Ca^2+^]_c_ and SPCa were measured in Fura2 loaded single fibers from mechanically isolated bundles of EDL muscles in vehicle-treated WT and *DMD*^*mdx*^ rats, and AAV2/9-MD treated *DMD*^*mdx*^ rats (MD-*DMD*^*mdx*^). Bars represent mean ± SEM values of [Ca^2+^]_c_ and SPCa in n EDL muscle fibers from N animals (n/N are indicated at the bottom of the bars). *: significantly different from mean value measured in WT muscle fibers; $: significantly different from mean value measured in Vehicle-*DMD*^*mdx*^ muscle fibers; One-way ANOVA and Fisher LSD post-hoc test, P < 0.05. **D** Maximal diaphragm contraction amplitude measured in vivo by echography. **E:** Whole muscle maximal tetanic tension measured in vitro in EDL from Vehicle-WT, Vehicle-*DMD*^*mdx*^ and MD-*DMD*^*mdx*^ rats. **F and G** Expression of TRPC3 mRNAs and proteins, measured by RTq-PCR and western blot, respectively, in EDL muscle extracts from Vehicle-WT, Vehicle-*DMD*^*mdx*^ and MD-*DMD*^*mdx*^ rats. **D–G** bars represent ± SEM values measured in N animals (N are noted at the bottom of the bars). *: significantly different from mean value measured in WT muscle; $: significantly different from mean value measured in Vehicle-*DMD*^*mdx*^ muscles; Kruskal–Wallis test and Conover-Iman post-hoc test, P < 0.05

One main objective of the present study was to determine whether TRPC3 could be a therapeutic target to sustain rAAV-MD treatment. In order to assess this hypothesis a series of experiments was conducted in 4 months old *DMD*^*mdx*^ rats that received systemic IV injections of a therapeutic dose of rAAV2/9-MD (3E13 vg/kg; MD-*DMD*^*mdx*^) at 1 month of age. Results obtained were compared to age-matched WT (Vehicle-WT) and *DMD*^*mdx*^ (Vehicle-*DMD*^*mdx*^) littermate rats treated in the same conditions conditions though injected with vector formulation buffer (vehicle). As previously observed in untreated animals, when compared to Vehicle-WT, EDL muscle of Vehicle-*DMD*^*mdx*^ rats exhibited fibers with higher resting [Ca^2+^]_c_ and SPCa, similar TRPC3 mRNA level and higher TRPC3 protein expression (Fig. 6B, C). In EDL and diaphragm muscles endogenous dystrophin was detected by western blot in Vehicle-WT rats but not in Vehicle-*DMD*^*mdx*^ nor MD-*DMD*^*mdx*^ animals (Fig. 6A). As expected, MD protein was highly expressed in EDL and diaphragm muscles of MD-*DMD*^*mdx*^ rats 3 months after rAAV2/9-MD injection (Fig. 6A). MD expression in EDL muscle was associated with a significant preservation of [Ca^2+^]_c_ and SPCa at the fiber level (Fig. 6B, C). Nevertheless, MD-associated preservation of Ca^2+^ homeostasis was only partial and the [Ca^2+^]_c_ and SPCa mean values were still significantly higher in MD-*DMD*^*mdx*^ EDLs compared to Vehicle-WT controls. We next assessed the impact of rAAV-MD injection on skeletal muscle function in vivo Diaphragm contraction amplitude was measured by ultrasound in the three groups of rats prior to sacrifice, together with in vitro EDL maximal isometric tension (Fig. 6D, E). When compared to Vehicle-WT counterparts, a significant decrease of diaphragm contraction amplitude and EDL maximal isometric tension were observed in Vehicle-*DMD*^*mdx*^ rats. In MD-*DMD*^*mdx*^ rats, diaphragm contraction amplitude was significantly preserved as compared to Vehicle-*DMD*^*mdx*^ animals, although it was still lower than that of Vehicle-WT rats (Fig. 6D). We confirmed by western blot that the diaphragm contraction amplitude preservation was associated with a significant MD expression in MD-*DMD*^*mdx*^ rats (Fig. 6A). Similar results were observed for EDL maximal isometric tension (Fig. 6E). Both parameters were significantly decreased in Vehicle-*DMD*^*mdx*^ rats compared to Vehicle-WT controls, and this was partially corrected by MD gene therapy (Fig. 6D, E). In parallel, the increase in TRPC3 protein levels was also partly prevented in MD-*DMD*^*mdx*^ EDL, without a significant change in TRPC3 mRNA (Fig. 6F, G).

## Discussion

The main aims of the present study were to assess the involvement of TRPC1 and TRPC3 channels in the DMD pathogenesis by participating in the Ca^2+^ homeostasis alterations taking place in the skeletal muscles. The experiments were conducted during the post-natal development of the *DMD*^*mdx*^ rat, an animal model that closely reproduces the human DMD disease with, in particular, a progressive and severe skeletal muscle necrosis and fibrosis, with significant reduction in muscle strength, and a decrease in spontaneous motor activity [27]. Considering that the rAAV-MD based treatments of DMD are currently promising but would rather lead to a milder BMD-like muscular dystrophy, we evaluated the potential of TRPC1 and TRPC3 to represent alternative or complementary therapeutic targets to rAAV-MD based treatments of DMD.

### The *DMD*^*mdx*^ rat model exhibits skeletal muscle calcium homeostasis alterations

Most of the pathogenesis and the preclinical studies concerning DMD were carried out in the *mdx* mouse. This animal model presents a much milder muscular dystrophic phenotype than human DMD patients [28]. This could be part of the reason for the poor translation of the findings achieved with these animals and this is why the *DMD*^*mdx*^ rat was generated [27]. The milder phenotype of the *mdx* mouse model probably depends on scale and cell proliferation differences allowing a better compensation of muscle fibers necrosis [44]. On the other hand, the cascade of cellular events induced by the lack of dystrophin expression leading to muscle fiber necrosis likely follows a similar scheme in mdx mice and DMD patients. In particular, Ca^2+^ alterations have been reported to be very early events in the DMD pathology. Calcium overload has been reported in muscle fibers of DMD boy fetus, measured in not fully differentiated human DMD myotubes and observed in the *mdx* mouse [16, 17, 31]. The mechanisms leading to a Ca^2+^ overload have been mostly deciphered in the *mdx* mouse due to the difficulty to work with fully differentiated living human cells. It has been shown that intracellular Ca^2+^ overload is mainly related to an increase of the SPCa through the accumulation of Ca^2+^ permeable ion channels [18]. One of the first aims of the present study was thus to assess whether the dystrophic phenotype of the *DMD*^*mdx*^ was associated with [Ca^2+^]_c_ and SPCa increases in skeletal muscle fibers. From 1.5 to 7 months of age, WT rats undergo puberty and reach adulthood [29]. Within this time window, we found that, in WT fast twitch muscle cells, the [Ca^2 +^]_c_ and SPCa values were not fixed and varied over time. The progressive increase of [Ca^2+^]_c_ and the parallel decrease of SPCa in WT EDL muscle fibers sustain the important role of Ca^2+^ homeostasis in EDL muscle fibers during post-natal development. One may be surprised by the apparent lack of correlation between the two processes. Nevertheless, SPCa is solely related to Ca^2+^ influx trough Ca^2+^ permeable channels whereas [Ca^2+^]_c_ is the snapshot of an equilibrium that depends not only on SPCa but also on numerous other processes, such as the balance between SR Ca^2+^ leaks and SR Ca^2+^ re-uptake, mitochondria or Ca^2+^-pumps and Na^+^/Ca^2+^ exchanger at the sarcolemma [45]. Interestingly, *DMD*^*mdx*^ rat model can experience MH-like episodes upon isoflurane-induced anesthesia that has forced us to further adapt anesthesia protocols when using this animal model. Similar MH-like episodes were also observed in some animals that were submitted to a long-lasting posture constraint, which was a source of stress for the animals. MH is a pharmacogenetic disorder that manifests as a hypermetabolic cascade initiated at the skeletal muscle cell mainly on exposure to halogenated anesthetics, like isoflurane [46]. Numerous MH like episodes have been reported in human patients after exposure to inhaled halogenated anesthetics including isoflurane, halothane, and sevoflurane [24], but that can be also triggered by stress. MH is mainly due to mutations in RYR1 and CACNA1S genes, that code for crucial Ca^2+^ channels, the ryanodine receptor from the sarcoplasmic reticulum and the Ca^2+^ voltage-gated channel subunit alpha1S located at the sarcolemma, respectively. No mutation in those genes have been reported in DMD patients exhibiting MH-like syndrome and although the underlying mechanisms are still not clear they likely depend on Ca^2+^ handling alterations [24]. MH-like events observed in the *DMD*^*mdx*^ rats were thus clues sustaining a Ca^2+^ homeostasis alteration in *DMD*^*mdx*^ skeletal muscle fibers. In order to further assess this hypothesis, we compared [Ca^2+^]_c_ and SPCa of EDL muscle fibers from the *DMD*^*mdx*^ rats to that of the WT ones. As early as 1.5 months of age, both characteristics were significantly higher in the *DMD*^*mdx*^ rats. In particular, the SPCa was found to be around the value of WT in *DMD*^*mdx*^ rat muscle fibers. From 1.5 to 7 months of age, [Ca^2+^]_c_ and SPCa progressively decreased in dystrophic muscle fibers as observed in WT muscle fibers, but were always higher in the *DMD*^*mdx*^ cells. These results demonstrated that in *DMD*^*mdx*^ rat skeletal muscle fibers Ca^2+^ homeostasis is altered with a higher Ca^2+^ influx trough the sarcolemma and intracellular Ca^2+^ overload.

### An increase in the TRPC3 protein accompanies early alterations in calcium homeostasis, while the TRPC1 channel exhibited late overexpression

One of the main objectives of the present work was to determine whether TRPC1 and TRPC3 channels could represent alternative or complementary therapeutic targets to rAAV-MD based treatments of DMD. TRPC1 protein expression was increased after 7 months of age in the dystrophic rats, when animals already exhibit a marked phenotype [27]. On the other hand, as early as 1.5 months of age, when the dystrophic phenotype is milder, *DMD*^*mdx*^ rat muscle TRPC3 protein expression was already twofold higher than in WT rats. Interestingly, in both genotypes TRPC3 protein level decreased and stabilized between 3 and 7 months of age. During this period of age, the expression of TRPC3 was still around twofold higher in dystrophic rats than in healthy ones. These results showed, firstly that TRPC3 plays an important role in the post-natal development of the rat EDL muscle, and secondly that TRPC3 is involved in the SPCa increase we observed in EDL muscle fibers from the *DMD*^*mdx*^ rat model. This is reinforced by the very similar evolution of the SPCa and TRPC3 protein expression in WT and *DMD*^*mdx*^ rats aged between 1.5 and 7 months. It is well documented that trafficking is a critical mode by which plasma membrane localization and surface expression of TRPC channels are regulated [47]. Thus, beside expression modification, involvement of TRPCs in SPCa increase may depend on TRPCs subcellular translocations from intracellular compartments. However, conflicting results have been obtained concerning the subcellular localization of TRPCs in striated muscles [48, 49]. Moreover, in striated muscle cells the plasma membrane is not restricted to the periphery but also forms intracellular invaginations called T-tubules. Therefore, in immunofluorescent confocal images, a channel revealed solely in the center of the cell could be expressed in the T-tubule walls and be directly involved in ion influx. In the present study, we focused our analysis on the comparison between WT and *DMD*^*mdx*^ muscle fibers. The objective was to determine if there was a difference between the 2 genotypes in the proportion of TRPC channels expressed at the level of the peripheral sarcolemma compared to the center of the cells. But we did not find any subcellular localization differences of TRPC1 nor TRPC3 between dystrophic and healthy EDL muscle fibers.

### SPCa increase is mainly related to TRPC3 channel activity in muscle fibers lacking dystrophin expression

Thus, SPCa increase may not be due to translocation of TRPC3 from intracellular vesicles to sarcolemma, but rather relies mainly on TRPC3 protein level and/or activity increases. The involvement of TRPC3 in SPCa was reinforced by the inhibition of divalent cation entrance by the specific inhibitor Pyr10 [33]. Although SPCa was more than twofold higher in dystrophic muscle fibers as compared to WT ones at 4 months of age, a similar SPCa proportion was inhibited in both genotypes. This indicated that the main part of SPCa increase in *DMD*^*mdx*^ rat muscle fibers was related to TRPC3. This leads us to propose that TRPC3 channels are involved in the pathogeneic process itself. This is reinforced by the noteworthy work published by Millay and colleagues in 2009, showing that overexpression of TRPC3 specifically in skeletal muscle induced muscular dystrophy in WT mouse [50]. Therefore, inhibition of TRPC3 activity and/or expression could minimize the dystrophic process in DMD.

### TRPC3 protein increase in* DMD*^*mdx*^ rat muscles likely relies on post-translational mechanisms

One striking result of the present study was that TRPC3 protein expression increased in *DMD*^*mdx*^ rat skeletal muscles whereas the level of the mRNA coding for this channel decreased, particularly at 1.5 months of age. Although further experiments are needed to clarify this apparent discrepancy, it may be explained by 2 main but nonexclusive hypotheses: (1) the expression of different isoforms that may be detected at the protein level but not at the mRNA level, and (2) the existence of post-translational protein changes that may modify TRPC3 turn-over. Importantly, the primers we used for RT-qPCR analysis were designed to amplify part of exon 2 of the TRPC3 mRNA from rat. This part of the unspliced TRPC3 mRNA is fully retrieved in predicted and isolated TRPC3 mRNA isoforms that have been reported up to now [36, 51, 52]. Similarly, the monoclonal antibody that was used in our study is directed to a peptide constituted of the first hundred amino acids in the N-terminal of the human TRPC3 protein. This part of the protein is highly conserved between humans and rats whatever the splicing of the coding mRNA. It is therefore likely that in the present study all the TRPC3 isoforms were measured, at both the mRNA and the protein levels. Our results clearly demonstrated that the *DMD*^*mdx*^ TRPC3 apparent MW (AMW) was 2 kDa higher than the WT one. This difference of AMW may be due to the expression of different TRPC3 isoforms. In the cerebellum from humans, guinea pigs, mice and rats, the team of Gary D Housley identified a short isoform of TRPC3 (TRPC3c), resulting from alternative splicing of exon 9 [36, 51]. They showed that TRPC3c mRNA was predominant in the cerebellum of these species as compared to the full TRPC3 mRNA (TRPC3b). Interestingly, recombinant TRPC3c and TRPC3b proteins expressed in HEK293 cells exhibited AMWs that were different about 3 to 4 kDa, a difference value of apparent AMW that is very closed to that we measured herein between *DMD*^*mdx*^ and WT. However, gel electrophoresis of the amplicon corresponding to the 8–10 exons showed that TRPC3 mRNA transcripts from *DMD*^*mdx*^ and WT rat muscles both conserved the exon 9. Thus, the difference in AMW of TRPC3 in *DMD*^*mdx*^ and WT muscles did not depend on exon 9 splicing. Moreover, we saw no difference between EDL TRPC3 whole cDNA *DMD*^*mdx*^ and WT rats, suggesting that the variation in AMW was not related to differential TRPC3 mRNA splicing.

Post-translational modifications, such glycosylation and phosphorylation, may influence the stability of proteins as well as their western blot AMW. One N-glycosylation site and several phosphorylation sites have been identified in TRPC3 protein [43]. Such modifications may influence TRPC3 stability, but they rather seem to regulate the channel basal activity [43]. Although further experiments are needed, the results obtained in the present study suggest that TRPC3 is N-glycosylated in healthy muscle rat fibers and un-glycosylated in the *DMD*^*mdx*^ ones. The higher AMW observed after the deglycosylation may be surprising at first glance, since one may expect a decrease in protein weight. Nevertheless, western blot protein separation not only depends on the protein size but also on its conformation. Indeed, it has been previously reported that adding N-glycosylation may induce a decrease in the AMW of a protein despite an increased molecular weight [53]. As TRPC3 unglycosylation has been reported to increase channel activity [54]. This supports the increased expression of TRPC3 and the subsequent dysregulations of Ca^2+^ homeostasis in EDL muscle fibers lacking dystrophin expression.

### rAAV-based MD only partially prevents Ca^2+^ homeostasis alterations, skeletal muscle force and TRPC3 protein increase

One key objective of the present study was to determine whether TRPC3 could be a therapeutic target for a complementary DMD treatment to rAAV-based MD therapy. In such a case, the incomplete correction of TRPC3 expression and activity by the rAAV-based MD therapy is a necessary condition to observe additive benefits. Gene transfer therapy based on rAAV-MD systemic delivery is a promising approach and clinical trials using this strategy are ongoing [12]. The MD transgene we used in the present study is very closely related to those used in the three ongoing clinical trials. For instance, both MD and the Sarepta micro-dystrophin transgenes contain N-terminus for binding to F-actin; spectrin repeats 1–3 and 24; hinges 1, 2, and 4; and the cysteine-rich domain [12]. Both transgenes use skeletal muscle specific promotors, and systemic injections of rAAV2/9.SP5.12-MD in the *DMD*^*mdx*^ rat used here, and rAAVrh74.MHCK7.micro-dystrophin, in humans for Sarepta clinical trials, resulted in a MD expression in 80–90% of skeletal muscle fibers. Therefore, it can be concluded that the rAAV-MD based treatment we used, is very close to those used in ongoing clinical trials. In the present study, we assessed for the first time the benefits of a rAAV-MD systemic delivery on the Ca^2+^ homeostasis in skeletal muscle fibers lacking dystrophin expression. As expected based on the intimate role of Ca^2+^ homeostasis alterations in DMD pathogenesis, we found that rAAV-MD systemic injections significantly counteracted [Ca^2+^]_c_ and SPCa in skeletal muscle fibers. Nevertheless, the benefit was only partial, and both [Ca^2+^]_c_ and SPCa remained at higher levels despite MD expression. Interestingly, similar results were observed when comparing TRPC3 expression level between WT skeletal muscle fibers, MD skeletal muscle fibers and skeletal muscle fibers lacking dystrophin. In particular, TRPC3 expression was still elevated in skeletal muscle fibers transfected by rAAV-MD. These results first reinforced the relation between DMD pathogenesis and TRPC3 expression alteration. They further suggest that pharmacological or molecular strategies dedicated to inhibit TRPC3 channel expression and/or activity could be effective after MD expression.

## Conclusion

In the present study, we demonstrated early increases of [Ca^2+^]_c_ and SPCa in the EDL fast-twitch muscles from *DMD*^*mdx*^ rats. This was accompanied by an increase in TRPC3 expression at the protein level. Finally, we showed that rAAV-MD based treatment induced a high MD expression level which was accompanied with significant but partial prevention of calcium homeostasis alterations, increase in skeletal muscle force and TRPC3 protein overexpression. These results show that correcting TRPC3 channel expression and/or activity appears to be a promising approach as a single or as a rAAV-based complementary therapy to treat DMD.

## Data Availability

The datasets used and/or analysed during the current study are available from the corresponding author on reasonable request.

## Abbreviations

[Ca^2^^+^]_c_: Cytosolic calcium concentration
AM: Acetyl-methyl ester
AMW: Apparent molecular weight
BMD: Becker muscular dystrophy
Cav-3: Caveoline 3
cDNA: Complementary DNA
DMD: Duchenne muscular dystrophy
EDL: *Extensor digitorum longus*
MD: Microdystrophin
MH: Malignant hyperthermia
mRNA: Messenger ribonucleic acid
NPS: Normal physiological solution
PBS: Phosphate buffer saline
PFA: Paraformaldehyde
Pyr10: N-(4-(3,5-bis(trifluoromethyl)-1H-pyrazole-1-yl)phenyl)-4- methylbenzenesulfonamide
rAAV: Recombinant adeno-associated virus
RT-qPCR: Reverse transcription quantitative polychain reaction
RyR: Ryanodine receptor
SPCa: Sarcolemmal permeability to calcium
SR: Sarcoplasmic reticulum
TRP: Transient receptor potential
TRPC: Canonical transient receptor potential
WT: Wild-type
